# Efficacy and Safety of High-Dose-Rate Brachytherapy of Single Implant with Two Fractions Combined with External Beam Radiotherapy for Hormone-Naïve Localized Prostate Cancer

**DOI:** 10.3390/cancers3033585

**Published:** 2011-09-14

**Authors:** Yasutaka Noda, Morio Sato, Shintaro Shirai, Kazushi Kishi, Takeshi Inagaki, Takeshi Mori, Isao Hara

**Affiliations:** 1 Department of Radiology, Wakayama Medical University, 811-1 Kimiidera, Wakayama Shi, Wakayama 641-0012, Japan; E-Mails: y-kiyose@wakayama-med.ac.jp (Y.N.); shshirai@wakayama-med.ac.jp (S.S.); kazushi.kishi@gmail.com (K.K.); 2 Department of Urology, Wakayama Medical University, 811-1 Kimiidera, Wakayama Shi, Wakayama 641-0012, Japan; E-Mails: tinagaki@wakayama-med.ac.jp (T.I.); tks-mori@wakayamah.rofuku.go.jp (T.M.); hara@wakayama-med.ac.jp (I.H.)

**Keywords:** prostate cancer, high dose rate brachytherapy, external beam radiation therapy, radical prostatectomy

## Abstract

The purpose of this study was to evaluate the efficacy and safety of high-dose-rate (HDR) brachytherapy of a single implant with two fractions plus external beam radiotherapy (EBRT) for hormone-naïve prostate cancer in comparison with radical prostatectomy. Of 150 patients with localized prostate cancer (T1c–T2c), 59 underwent HDR brachytherapy plus EBRT, and 91 received radical prostatectomy. The median follow-up of patients was 62 months for HDR brachytherapy plus EBRT, and 64 months for radical prostatectomy. In patient backgrounds between the two cohorts, the frequency of T2b plus T2c was greater in HDR brachytherapy cohort than in prostatectomy cohort (27% versus 12%, p = 0.029). Patients in HDR brachytherapy cohort first underwent 3D conformal RT with four beams to the prostate to an isocentric dose of 50 Gy in 25 fractions and then, a total of 15–18 Gy in two fractions at least 5 hours apart. We prescribed 9 Gy/fraction for target (prostate gland plus 3 mm lateral outside margin and seminal vesicle) using CT image method for radiation planning. The total biochemical failure-free control rates (BF-FCR) at 3 and 5 years for the HDR brachytherapy cohort, and for the prostatectomy cohort were 92% and 85%, and 72% and 72%, respectively (significant difference, p = 0.0012). The 3-and 5-year BF-FCR in the HDR brachytherapy cohort and in the prostatectomy cohort by risk group was 100 and 100%, and 80 and 80%, respectively, for the low-risk group (p = 0.1418); 92 and 92%, 73 and 73%, respectively, for the intermediate-risk group (p = 0.0492); and 94 and 72%, 45 and 45%, respectively, for the high-risk group (p = 0.0073). After HDR brachytherapy plus EBRT, no patient experienced Grade 2 or greater genitourinay toxicity. The rate of late Grade 1 and 2 GI toxicity was 6% (n = 4). No patient experienced Grade 3 GI toxicity. HDR brachytherapy plus EBRT is useful for treating patients with hormone-naïve localized prostate cancer, and has low GU and GI toxicities.

## 1. High Dose Rate (HDR) Brachytherapy

Brachytherapy for localized prostate cancer is classified into low-dose-rate (LDR) and high-dose-rate (HDR) brachytherapies. The term ‘brachytherapy,’ however, is sometimes used as a synonym for LDR brachytherapy [1–4], despite the history of HDR brachytherapy. HDR brachytherapy utilizes γ-rays (Ir-192) to irradiate the whole prostate gland. The prostate gland is punctured by an applicator needle and a small radioactive source of Ir-192 is inserted through the applicator by remote control. Accordingly, the operator is not exposed to radiation, and the patient does not receive continuous irradiation after discharge because the radioactive source is retrieved immediately after treatment. Based on information regarding the position and number of applicators and the clinical target volume of the prostate gland, radiation oncologists use a computer to formulate a treatment plan that delivers homogeneous dose distribution to cover the whole prostate gland. Thereafter, exposure time is computed using an isodose plot to create the radiation treatment plan. HDR brachytherapy can be undertaken not only for low-risk grade but also for intermediate- and high-risk grades of prostate cancer, by increasing the radiation dose per fraction.

According to the HDR brachytherapy administration pattern, multiple implants with a single fraction or a single implant with multiple fractions are available. Stromberg *et al.* initiated the use of three implants with a single fraction of 5.5 Gy each [5], while Mate *et al.* used a single implant with four HDR fractions of 3–4 Gy each [6], and Martinez *et al.* reported promising results using two or three implants with a single fraction of 5.5–11.5 Gy each [7]. Yoshioka *et al.* reported a single implant with eight fractions of 5.5 Gy each for 5 days as monotherapy [8]. Hiratsuka *et al.* reported promising long-term biochemical control rates using a single implant with four fractions of 5.5 Gy each for 2 days [9]. In a single implant with multiple fractions, the applicators remain in place for all fractions and the patient maintains the dorsal lithotomy position during the fractions.

Martinez *et al.* achieved the promising outcome of controlling PSA for the patients even in the high risk group using HDR brachytherapy of two or three implants with a single fraction of 5.5 to 11.5 Gy each. The single fraction of multiple implants can be done within a half day but its weakness is the repeated lumbar anesthesia and treatment planning every one or two weeks [6]. Meanwhile, the main reason why HDR brachytherapy with a single implant with multiple fractions has not prevailed worldwide is that the patient is required to remain immobilized for one to five days in the supine position with the bilateral lower extremities raised. However, if HDR brachytherapy could be completed using a single implant with two fractions a day, the burden to the patient would be greatly reduced [10] and it delivers the same physical burden per implant as HDR brachytherapy of multiple implants with a single fraction. At our institute, lumbar anesthesia is initiated at 8:30 am, and the treatment time during which the lithotomy position must be maintained is from 9 am to 5 pm. The patient begins to feel discomfort in this position for approximately the last 3 hours of treatment with the decreasing efficacy of the lumbar anesthesia after 2 pm. The burden to the patient in the present clinical study is less than that in previous studies of HDR brachytherapy because our HDR brachytherapy is completed in half a day, which is almost the same burden as for LDR brachytherapy. Forα/β values of 1.2, 5 and 10 for tumor control probability, biological effective doses using the present HDR brachytherapy of 15 Gy or 18 Gy with external beam radiation therapy (EBRT) of 50 Gy were 90.7 Gy or 107.3 Gy, 76.8 Gy or 86 Gy and 71.9 Gy or 78.5 Gy. The lowα/β ratios for prostate cancer control probability are currently accepted and make hypofractionated HDR brachytherapy ideal.

## 2. Method for HDR Brachytherapy

**Figure 1.**
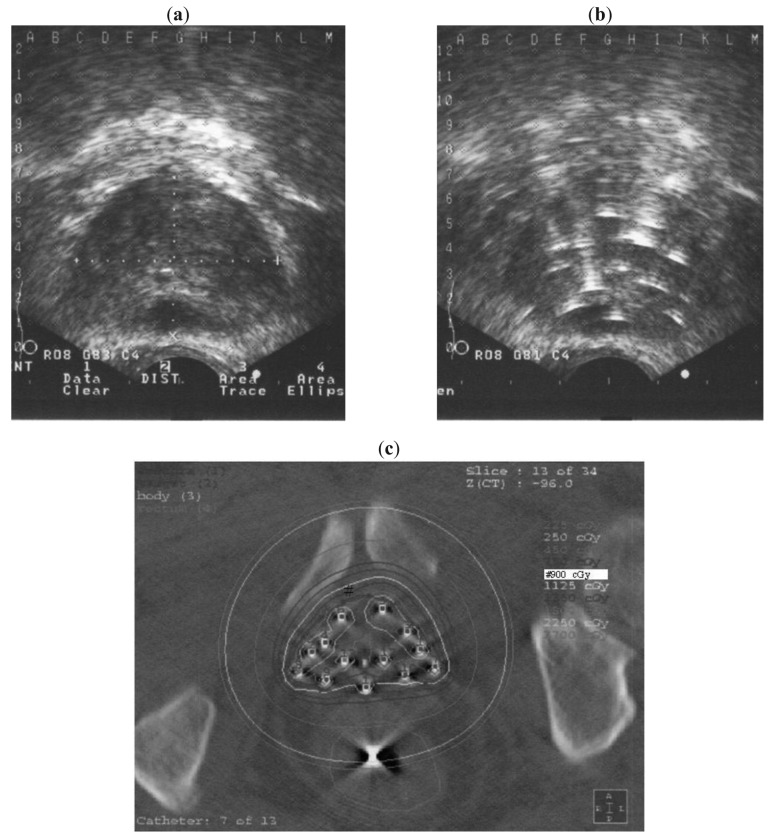
Axial trans-rectal ultrasound (TRUS) showing the axial largest cross-section of the prostate gland before (**a**) and after (**b**) insertion of applicator needles. (**c**) Axial view after computed tomography-based treatment planning reveals homogeneous radiation distribution to the whole prostate gland.

Brachytherapy implantation was conducted under lumbar anesthesia and with intravenous antibiotic cover. A urinary catheter was inserted, with the balloon inflated with saline and positioned at the bladder neck to aid localization. In preparation for lumbar anesthesia, patients who usually take anticoagulants (e.g., aspirin) were ordered not to take this medication for 1 week before HDR brachytherapy. Intravenous anesthesia was used for patients contra-indicated for lumbar anesthesia because of the risk of ceasing anticoagulants and those with highly deformative lumbar spondylosis. Each patient was positioned in the dorsal lithotomy position. The template was pressed firmly and directly against the perineal skin. Transrectal ultrasound (TRUS) was performed to enable the prostate to be viewed in the axial and sagittal planes and to measure its volume. The X-Y axis coordinates (A to M, 1 to 12, Figure 1a) were recorded on the template and the corresponding X–Y axis coordinate image was projected onto the axial TRUS image. Prior to needle placement, the probe was positioned to encompass the largest axial section of the prostate under TRUS guidance, and the contour of the prostate was outlined as a reference plane.

We aimed to place the applicator needles in the largest section of the prostate. For safety, in the first piercing with the applicator needle we targeted the prostate parenchyma in the periphery, avoiding the urethra. Under sagittal view real-time TRUS, we generally checked the position and direction of the urethra prior to piercing; each applicator needle pierced the whole prostate, after which the length from the tip of the needle to the base of the prostate was measured. After the initial two or three piercings, the prostate commonly moves right or left, up or down, or rotates; therefore, the positions of the urethra and the needle were reconfirmed after each needle placement using axial view real-time TRUS. In most cases, positioning was adjusted from the initial reference plane as necessary. Symmetric right and left piercing helps prevent movement of the prostate gland. Applicator needles and applicator stoppers (Trocar Point Needles and Needle Stoppers; Nucletron, Veenendaal, The Netherlands) and a template with a coverage plate (Toiseimedical, Osaka, Japan) were used to stabilize and guide the insertions.

The applicator needles were distributed evenly throughout most of the prostate. Because invasion of prostate cancer to the seminal vesicles at the microscopic level cannot be ruled out even in the localized group, any needle that was positioned touching a seminal vesicle was advanced to pierce it. Needles were inserted by urologists under the guidance of radiation oncologists. Between 11 and 18 needles were utilized depending on the size of the prostate [Figure 1b]. Following needle placement, the patient was taken to the simulation room.

## 3. Treatment Plan for HDR Brachytherapy

Treatment plans for HDR brachytherapy were created using the X-ray film method in 45 cases [9-11], and using CT image method in nine cases (Figure 1c).

## 4. X-ray Film Method

Treatment volume was cylindrical, extending from the base to the apex, with the equivalent to the reference plane. A dummy source was packed in each needle. Radiographs of the two oblique views were obtained to identify the location the location of all applicator needles and the rectal dose calculator. These locations and measurements were entered into the computer for radiation treatment planning. A real-time rectal dosimeter (AB Uppsala type, IDF-5; Scanditronix Wallhofer, Stockholm, Sweden) comprising five sensors was inserted into the rectum to survey the rectal dose prior to CT. Doses at 3, 5, and 10 mm from the anterior rectal wall from the sensor nearest the prostate were estimated during treatment. The tip of the dosimeter was placed beyond the tip of the applicator needles.

## 5. CT Image Method

Injection of contrast medium to the urethral catheter was conducted prior to CT to check for urethral injury, measurement of anterior rectal wall thickness, and any movement of the applicator needles. CT with 3-mm slices was performed for radiation planning. The prostate gland was identified with reference to the length from the tip of the needle to the base, as measured by TRUS: the base slice (caudal edge) image of the prostate gland can be identified on CT by counting 3-mm thickness slice from needle tip image regarding the longest length of the applicator.

As for the area for prescription doses, we contoured the margin of the prostate gland plus 5 mm lateral margin and seminal vesicle as the treatment target. However, as for the base portion (rectum side), the treatment target margin was equivalent to the prostate gland itself without margin. The rectum and urethra were also contoured as risk organs. This information was entered into a computer for treatment planning.

## 6. Treatment Plan

**Figure 2.**
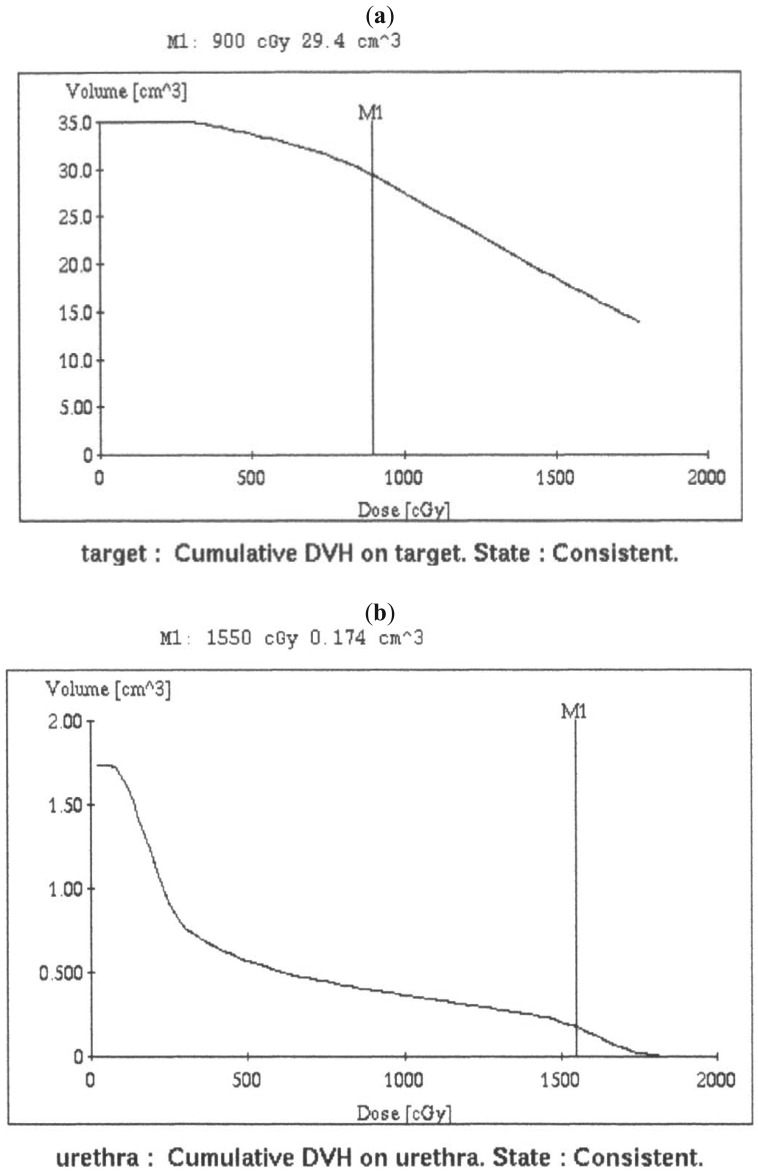

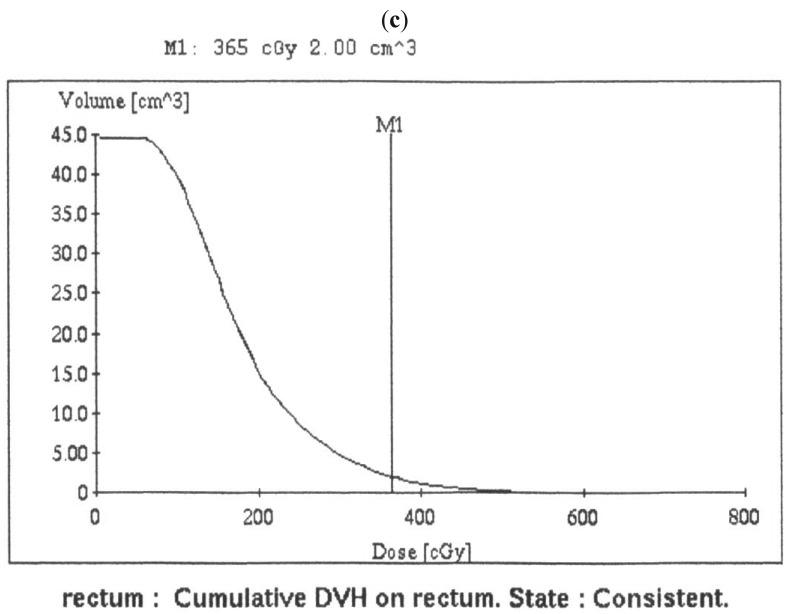
(**a**) The treatment plan was performed using CT image method. The prescription dose for the target was 9 Gy. Cumulative dose volume histogram (DVH) for the target (M1 = V100, 900 cGy irradiated to 29.4 cm^3^ of whole prostate gland plus 3 mm margin and seminal vesicle for prostate gland volume, 21 cm^3^); (**b**) Cumulative DVH for the urethra (M1 = D10: 1550 cGy, the maximum radiation dose to 10% of urethral volume, 1.74 cm^3^); and (**c**) DVH for the rectum (M1 = D_2 mL_: 365 cGy, the maximum radiation dose to 2 mL of the total rectal volume). (Dear author, please mention this FIGURE 2 somewhere in the body text. Please also provide higher resolution figures.)

The treatment plan was conducted *via* Plato (Nucletron), which enabled us to identify the irradiation time for each applicator needle and to create isodose plots in the axial, sagittal, and coronal planes. The treatment plan was generated using standard geometric optimization in both X ray method and CT image method. The treatment plan in CT image method was further revised to lessen rectal doses with geographic optimization. All HDR fractions were performed using the microSelectron-HDR ^192^I-remote afterloading system (Nucletron). Dwell positions were activated at intervals of 2.5 mm along each applicator. A total of 15–18 Gy in two fractions was prescribed at least 5 hours apart [10,11]. When we prescribed 9 Gy/fraction for target using CT image method for radiation planning, median V100, volume of prostate gland, V100/prostate gland volume, urethra D10 and D_2 mL_ were 29.9 cm^3^ (17.8–40.8 cm^3^), 24.1 cm^3^ (15.4–40.1 cm^3^), 1.24 (1.05–1.42), 1677 cGy (1307–2488 cGy) and 348 cGy (294–395 cGy), respectively (Figure 2).

Forty cases and nineteen cases received 15 Gy and 18 Gy, respectively. The next 26 patients received HDR brachytherapy of 7.5 Gy per fraction and CT with 3 mm slices before 1^st^ and 2^nd^ brachytherapy to explore the relation rectal dose and thickness of rectal anterior wall. Maximal surveyed dose to rectal surface during the 1^st^ and 2^nd^ HDR brachytherapy was 188 ± 51 cGy and 220 ± 35 cGy respectively(P < 0.001). Thickness of anterior rectal wall before 1^st^ and 2^nd^ HDR brachytherapy was 8.78 ± 4.34 mm and 14.95 ± 4.09 mm (P < 0.001) respectively [11]. In this experience, there was up to 7 mm of movement of the applicator needle between the first and second HDR brachytherapy probably because of swelling of the prostate gland.

The applicators and template were removed immediately after the second fraction. The entire process from initiating spinal anesthesia for the insertion of applicator needles to completing the second fraction required at most 8 to 9 hours, from 8:30 am to 5:30 pm. Patients were discharged the following day and placed on oral antibiotics for one week.

## 7. EBRT

Every patient started EBRT before brachytherapy. CT images of the prostate were acquired in a 3D planning system (Pinnacle; ADAC Laboratories, Milpitas, CA, USA) using a CT scanner (CTS-ZOSPH; Shimadzu, Kyoto, Japan) to obtain 3-mm-thick slices. Patients with T1c–T2c prostate cancer underwent 3D conformal RT with four beams to the prostate to an isocentric dose of 50 Gy in 25 fractions using 10 MV photons. Gross tumor volume (GTV) was defined as the entire prostate and the seminal vesicles; the clinical target volume (CTV) comprised the GTV plus 5 mm in all directions [10,11].

## 8. Follow-Up Study

For patients who received HDR brachytherapy plus EBRT, prostate-specific antigen (PSA) and morbidity data were checked by both urologists and radiation oncologists; patients who received radical prostatectomy were checked by urologists. Checks were performed at intervals of one month post-HDR brachytherapy and then every three months thereafter. If an increase in PSA was detected, PSA was rechecked after a 1-month interval. The American Society of Therapeutic Radiology and Oncology (ASTRO) definition for biochemical failure was listed in 1997 [12]. ASTRO biochemical failure occurs if a patient has three consecutive rises in serum PSA. The biochemical failure data were backdated to midway between the nadir and the first PSA rise. Thereafter, the Radiation Therapy Oncology Group (RTOG)-ASTRO Phoenix Consensus Conference recommended in 2006 that the biochemical failure-free control rate (BF-FCR) date should be listed as two years short of the median follow-up. Accordingly, This study was conducted to choose to quote data using the Phoenix definition of absolute nadir + 2 ng/mL at call, without backdating [13].

Genitourinary, gastrointestinal, and adverse sexual effects related to HDR brachytherapy with EBRT were evaluated every three months and graded according to Common Terminology Criteria for Adverse Events v3.0 (CTCAE v3.0). Sexual morbidity was assessed by direct questioning of patients regarding erection and ejaculation.

## 9. Safety Assurance for HDR Brachytherapy

In radiation treatment for prostate cancer, the risk organs are the rectum and the urethra adjacent to the prostate gland. The irradiated dose to the rectum is predicted in the treatment plan, but predicted dose to the rectum does not always reflect the real dose. The position of the prostate gland varies with the volume of rectal content: the anterior wall of the rectum comes closer to the prostate gland as the content increases. Moreover, insertion of applicator needles into the prostate gland generates edema, forcing the prostate gland closer to the rectum and resulting in dose escalation to the rectum. Accordingly, the data for predicted dose in a treatment plan is not always accurate. A previous study found an increase in surveyed rectal doses over time: the maximum surveyed rectal doses during the 1^st^ HDR brachytherapy one hour after radiation planning were 1.1 times greater than the estimated doses, and the maximum surveyed doses during the 2^nd^ HDR brachytherapy five hours later were 1.2 to 1.3 times greater than that during the 1^st^ HDR brachytherapy [11]. Although we have no experience in surveying following-day rectal doses, HDR brachytherapy the following day is considered to cause an increase in rectal dose that exceeds that calculated as the estimated dose. We have accumulated data regarding surveyed rectal doses during 1^st^ and 2^nd^ HDR brachytherapy. Using these data, we could estimate the maximum real rectal dose during the 2^nd^ HDR brachytherapy from the surveyed rectal doses of the 1^st^ HDR brachytherapy.

At our institute, we attempt to increase the dose per fraction in HDR brachytherapy; to avoid adverse effects to the rectum, we predict the maximum rectal dose during the 2^nd^ HDR brachytherapy using the surveyed doses during the 1^st^ brachytherapy, and can regulate the prescribed doses of 2^nd^ HDR brachytherapy accordingly, so that the total dose to the rectum does not exceed 8 Gy. Even if the dose per fraction increased from 7.5 Gy to 9 Gy, our data surveying rectal doses following total dose irradiation (15 to 18 Gy) did not reveal to exceed 8 Gy to the rectum. We prescribe 50 Gy of 3D conformal radiotherapy (3-DCRT) of external beam radiation treatment to the whole prostate gland and seminar vesicles, which we regulate not to exceed 58 Gy of the total real maximum rectal dose. In previous reports of adverse effects to the rectum, tolerant doses to the rectum were 70 Gy or more, and doses of 60 Gy or less to the rectum were viewed as safe rectal doses [14].

Urethral stricture is another late adverse effect of concern. In LDR brachytherapy, excess irradiation to the urethra is considered the cause of urethral stricture. To avoid this effect, it is recommended that radioactive particles should not be placed adjacent to the urethra, but rather around the periphery of the urethra. There are several reports regarding urinary stricture in LDR and HDR brachytherapy, and the insertion of applicators to the area adjacent to that surrounding the urethra tends to be avoided. Martinez et al. reported urinary stricture in 12% of subjects with three implants and 2% with two implants following HDR brachytherapy [7,15]. Urethral stricture may be caused by injury and irradiation to the urethra during insertion of the applicator needle. To avoid injuring the urethra, at our institute we usually insert the applicators under trans-rectal ultrasound (TRUS) with the consent with the urologists, and obtain CT with urethral injection of contrast medium to identify the position of applicator needles with respect to the urethra and to confirm that the applicators do not injure the urethra.

## 10. Acute and Chronic Adverse Effects

In terms of GU effects after brachytherapy, almost all patients experienced macroscopic hematuria and urinary urgency. Macroscopic hematuria generally resolved within one week. This symptom was probably caused by needle insertion or urinary balloon insertion, but not by RT. Urinary urgency, including an increase in urinary frequency in the daytime and nocturia were the main symptoms after RT. However, the urinary urgency showed improvement within one month, and was largely resolved three months later. The rate of Grade 1 to 2 GU toxicity was 25% (n = 30) at one month. One patient had Grade 3 GU toxicity requiring balloon insertion to the urinary bladder because of bladder tamponade. This patient had a past history of balloon insertion for prostatic hypertrophy. This symptom decreased over time. No patient experienced Grade 2 or greater GU toxicity at six months or later. Urinary stricture is known as Grade 3 late GU toxicity, and is caused by insertion of the applicator needle and by irradiation of the urethra; thus, it is important that the applicator needle does not pierce the urethra. None of our patients with a single implant experienced the adverse effect of urethral stricture of late genitourinary (GU) Grade 3 [10].

With regard to GI toxicity over time, the main symptom after RT was diarrhea, which was largely resolved within one month. The rate of acute Grade 1 and 2 GI toxicity was 7% (n = 8) at one month. Four patients experienced spotty rectal bleeding 12–24 months after RT. The rate of late Grade 1 and 2 GI toxicity was 6% (n = 4). Colonic endoscopy revealed rectal varicosity in two patients, rectal polyp in one, and vascular enlargement in one. The patient with vascular enlargement underwent biopsy of the anterior rectal wall and rectal bleeding persisted from the biopsy site. Symptoms improved after receiving a steroid enema. There was no Grade 3 GI toxicity at any point. The frequency of late GU and GI adverse effects in the present treatment is among the lowest of those reported [14,16,17].

We investigated changes in sexual function over time. The disorder rates for erection and ejaculation showed an increase over time. The sexual function preservation rate decreased to 35 and 25% at three and five years, respectively, after treatment.

## 11. Biochemical and Clinical Outcome

This two-arm study is a retrospectively comparison in a single institute. All patients who received HDR brachytherapy plus EBRT or radical prostectomy were examined by the referring urologists, who conducted an initial evaluation that included medical history, physical examination, serum PSA measurement, and histological examination. Tumor stage for both cohorts of HDR brachytherapy and PR was determined with rectal digital palpitation of our urologists. Urologists at our hospital did not use hormone therapy as the first option of treatment for radical prostatectomy and HDR brachytherapy + EBRT, and reserved hormone therapy as a final option after relapse. Of the 150 patients with localized prostate cancer (T1c–T2c) who were treated in our hospital from 2000 to 2004, 59 underwent HDR brachytherapy and EBRT, and 91 received radical prostatectomy. The patients made choice of the treatment based on the information from urologists. The analysis was conducted in 2008. The median follow-up was 62 months (48–108 months) for patients treated by HDR brachytherapy and EBRT, and 64 months (42–112 months) for patients treated by radical prostatectomy. The biochemical outcomes in the two cohorts were explored retrospectively. Patient backgrounds (age, T stage, Gleason score, PSA level prior to treatment) between the two cohorts are shown in Table 1. Except for T stage, there was no significant difference in patient backgrounds between the two cohorts. In short, the frequency of T2b plus T2c was greater in HDR brachytherapy cohort than in prostatectomy cohort (27% versus 12%, p = 0.029). Patients were assigned to a risk group based on pretreatment variables that independently affected the PSA failure-free survival. The parameters included pretreatment PSA ≤ 10 ng/mL, T stage T1c–T2a, and Gleason score ≤ 6. When all three indicators were present, the patient was assigned to a low-risk group. In the case of an increase in the value of one of the indicators, the patient was assigned to an intermediate-risk group; for an increase in two or more indicators, the patient assigned to a high-risk group.

**Figure 3.**
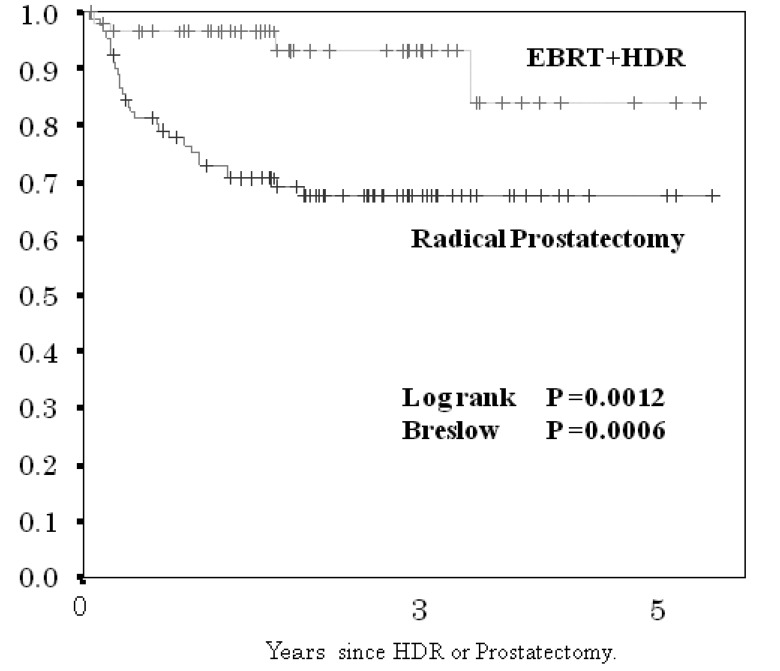
Comparison of biochemical failure-free control rate between 59 patients treated by HDR + EBRT and 91 patients treated by radical prostatectomy.

Biochemical failure-free control rate (BF-FCR) at three and five years for the HDR brachytherapy plus EBRT cohort, and for the radical prostatectomy cohort were 92 and 85%, and 72 and 72%, respectively (significant difference, p < 0.0012; Figure 3).

**Figure 4.**
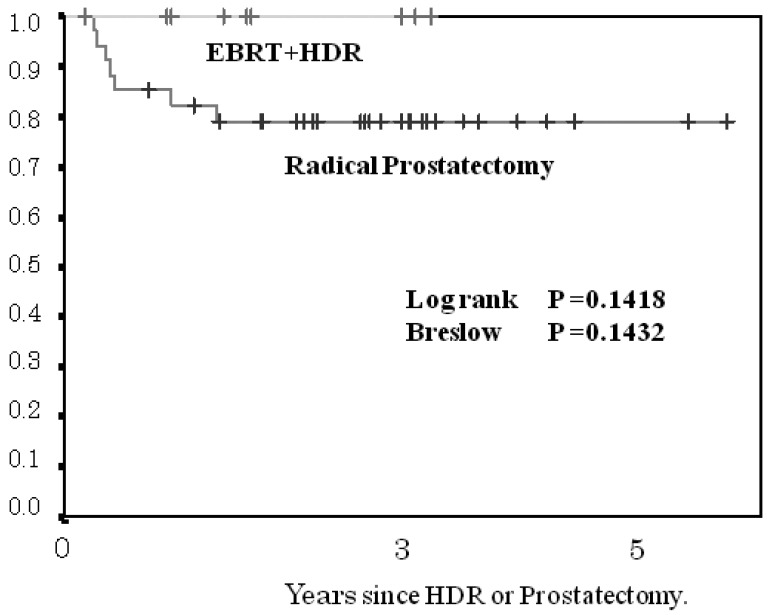
Comparison of biochemical failure-free control rate between the low-risk group treated by HDR + EBRT and patients treated by radical prostatectomy.

**Figure 5.**
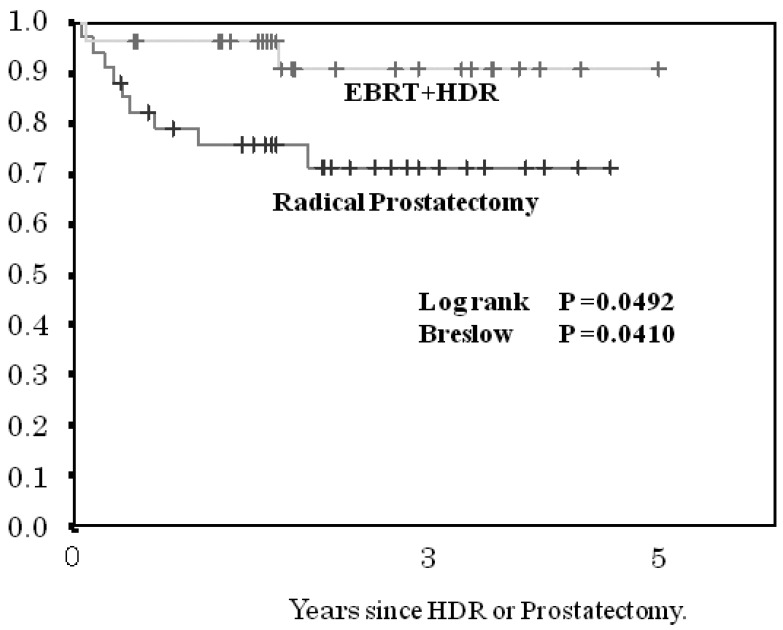
Comparison of biochemical failure-free control rate between the intermediate-risk group treated by HDR + EBRT and patients treated by radical prostatectomy.

**Figure 6.**
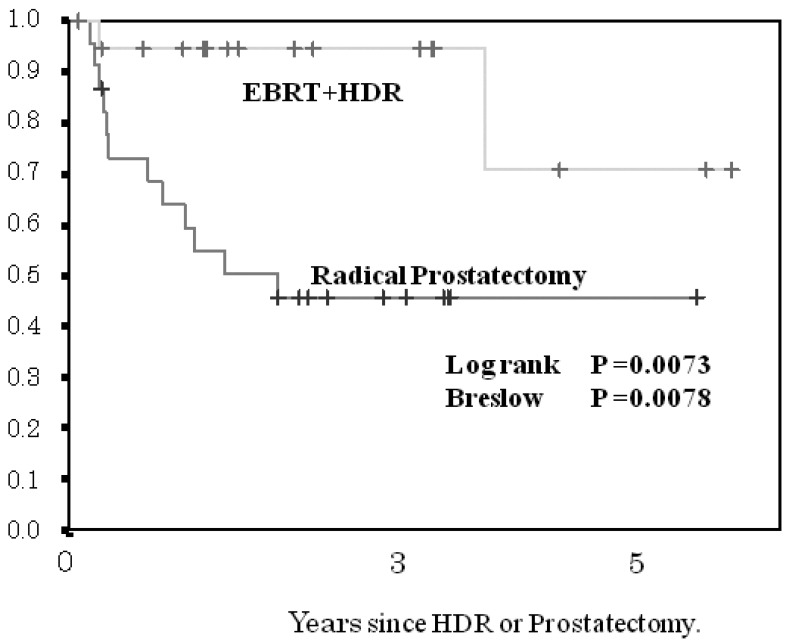
Comparison of biochemical failure-free control rate (BF-FCR) between the high-risk group treated by HDR + EBRT and patients treated by radical prostatectomy.

The 3- and 5-year BF-FCR in the HDR brachytherapy cohort and in the prostatectomy cohort according to risk group was 100 and 100%, and 80 and 80%, respectively, for the low-risk group (p < 0.1418, Figure 4); 92 and 92%, 73 and 73%, respectively, for the intermediate-risk subgroup (p < 0.0492, Figure 5); and 94 and 72%, 45 and 45%, respectively, for the high-risk subgroup (p < 0.0073, Figure 6). The patients with PSA failure after HDR brachytherapy plus EBRT received hormone therapy and those after radical prostatectomy received post-operative three-dimensional radiotherapy.

Multivariate analysis using logistic regression and Cox proportional hazard regression between the two cohorts was conducted with respect to age, Gleason score, T stage, pretreatment PSA value, and therapy. Pretreatment PSA value and mode of therapy were significant factors differentiating BF-FCR between the two cohorts (Table 2).

Previous studies have reported no significant difference in biochemical failure for patients in the low-risk group between surgical prostatectomy and LDR brachytherapy [16,17]. In the present study, no significant difference was observed in the low-risk group for BF-FCR between the HDR brachytherapy plus EBRT and the radical prostatectomy cohorts. The significant difference in BF-FCR for the intermediate- and high-risk groups was observed between the HDR brachytherapy plus EBRT and the radical prostatectomy cohorts. These results were impressive for us radiologists but not for urologists and then, the publication was hesitated at that time. However, these results were merely created in single institute and a retrospective study. Then, the further examination for a prospective study in multiple institutes has to be attempted to conclude.

Following the results of this study, the urologists at our hospital have adopted endoscopic radical prostatectomy with hormone therapy prior to surgery, and also continue to support HDR brachytherapy plus EBRT for intermediate- and high-risk patients.

## 12. Combination of HDR Brachytherapy with Hormone Therapy

Hormone therapy alone generally leads to a decrease in PSA values; the sensitivity to hormones gradually decreases over time, with the rate of PSA failure increasing a few years later. Therefore hormone therapy alone is not recommended to be the first-line treatment for patients with localized prostate cancer because of the availability of radical prostatectomy, or radiation treatment to prolong survival [13,18]. The combination of hormone therapy and radiotherapy is conducted worldwide for high risk group patients with prostate cancer [19,20]. When these two treatments are combined in this manner, the efficacy of radiotherapy is unclear, and the masking effect of hormone therapy makes the contribution of radiotherapy unclear. Facial flushing and thrombosis are known as early side effects of hormone therapy, while thrombosis, osteoporosis, and ulcers are late side effects [21-23]. In the present study, hormone-naïve patients were treated with HDR brachytherapy and EBRT, and hormone therapy was initiated when patients had PSA failure following brachytherapy.

Massive hemorrhage during prostatectomy, and urinary incontinence, and permanent impotence following surgical prostatectomy are known to be encountered, but these acute adverse effects were least observed in the HDR brachytherapy cohort. The therapeutic efficacy of HDR brachytherapy plus EBRT for localized prostate cancer was satisfactory in terms of the PSA failure rate. The weakness of the present study is the prolonged treatment time necessary to deliver EBRT in addition to HDR brachytherapy. Nevertheless, we consider that the present treatment is an acceptable minimally invasive treatment because it has a low incidence of adverse effects.

## 13. Conclusions

Although the present study merely confirmed the efficacy and safety of minimal invasiveness of HDR brachytherapy with a single implant with two fractions in the limited cases, we are encouraged to explore methods of enhancing therapeutic effect while maintaining safety. We intend to supply the dose escalation per fraction to 9 Gy or more, in the treatment of intermediate-risk, high-risk, and advanced (T3a or T3b) groups.

## Figures and Tables

**Table 1.** Comparison of patient characteristics between EBRT+HDR-B and radical prostatectomy.

**age(years)**	**EBRT+HDR-B**	**Radical prostatectomy**	**P value**
≦70	16 (27%)	39 (43%)	0.058

>70	43 (73%)	52 (57%)	
T stage			
T1c+T2a	43 (73%)	80 (88%)	0.029

T2b+T2c	16 (27%)	11 (12%)	
Gleason score			
≦6	35 (59%)	59 (65%)	0.604

7≦	24 (41%)	32 (35%)	
Pre-treatment PSA (ng/mL)			
≦10	25 (42%)	47 (52%)	0.316
>10	34 (58%)	44 (48%)	

Note: T = tumor, PSA = prostate specific antigen. EBRT = external beam radiation therapy. HDR-B = high-dose-rate brachytherapy.

**Table 2.** Multivariate analysis.

	**Logistic regression**		**Cox proportional hazard regression**	

	**P**	**Odds ratio**	**P**	**Odds ratio**
Age(years)	0.440	1.442	0.302	1.492
Gleason score	0.752	0.861	0.968	0.985
T stage	0.981	1.015	0.909	1.057
Pre-treatment PSA (ng/mL)	0.002	4.554	0.004	3.445
Therapy	<0.0001	8.398	0.001	6.042
